# *rac*-2-Phenyl-1-[(2,4,6-triiso­propyl­benzene)­sulfon­yl]aziridine

**DOI:** 10.1107/S1600536814000257

**Published:** 2014-01-15

**Authors:** Christopher Golz, Hans Preut, Carsten Strohmann

**Affiliations:** a Fakultät für Chemie und Chemische Biologie, Technische Universität Dortmund, Otto-Hahn-Strasse 6, 44221 Dortmund, Germany

## Abstract

In the title compound, C_23_H_31_NO_2_S, the geometry of the triiso­propyl­phenyl group is slightly distorted, with elongated C—C bonds at the *ipso*-C atom, and an S atom which deviates from the benzene ring plane by 0.228 (2) Å. This distortion is caused by the bulky substituents and, in comparison, an unbent geometry is observed in *N*-toluene­sulfonyl­aziridine [Zhu *et al.* (2006 ▶). *Acta Cryst.* E**62**, o1507–o1508]. π–π inter­actions between adjacent benzene rings [centroid–centroid distance = 3.7928 (11) Å] and are observed.

## Related literature   

For structures containing the triiso­propyl­benzene­sulfonyl group with detailed discussion of the geometry, see: Sandrock *et al.* (2004 ▶); Laba *et al.* (2009 ▶). For the li­thia­tion of activated aziridines, see: Huang *et al.* (2009 ▶) and for a general review on aziridinylanions, see: Florio & Luisi (2010 ▶). For the most recent synthesis of the title compound, see: Kavanagh *et al.* (2013 ▶). For deprotonation reactions of aziridinyl anions to amines, see: Gessner & Strohmann (2007 ▶, 2008*a* ▶,*b* ▶); Unkelbach *et al.* (2012 ▶).
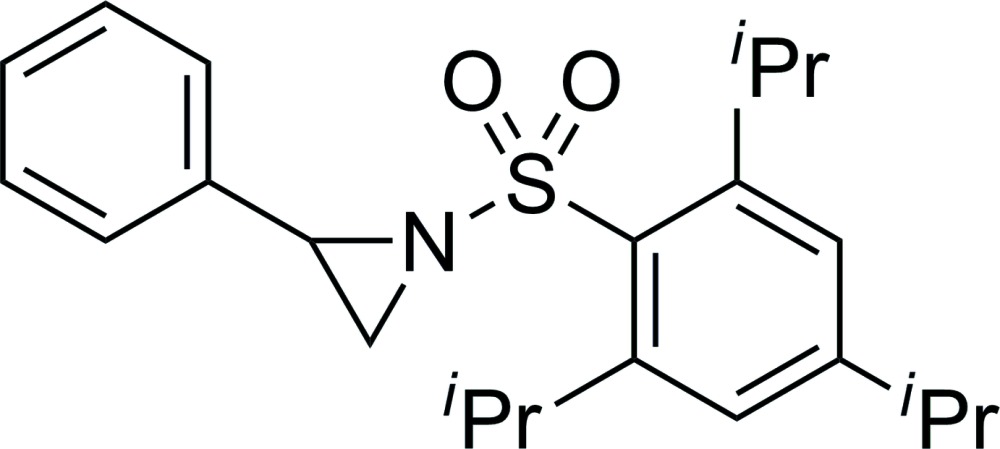


## Experimental   

### 

#### Crystal data  

C_23_H_31_NO_2_S
*M* *_r_* = 385.55
Triclinic, 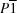

*a* = 6.3037 (3) Å
*b* = 9.6995 (5) Å
*c* = 18.6675 (9) Å
α = 75.280 (4)°
β = 86.842 (4)°
γ = 84.404 (4)°
*V* = 1098.11 (10) Å^3^
*Z* = 2
Mo *K*α radiation
μ = 0.16 mm^−1^
*T* = 173 K
0.31 × 0.05 × 0.04 mm

#### Data collection  

Agilent Xcalibur Sapphire3 diffractometer
Absorption correction: multi-scan (*CrysAlis PRO*; Oxford Diffraction, 2012 ▶) *T*_min_ = 0.952, *T*_max_ = 1.000
17550 measured reflections
4314 independent reflections
3633 reflections with *I* > 2σ(*I*)
*R*_int_ = 0.045

#### Refinement  

*R*[*F* ^2^ > 2σ(*F* ^2^)] = 0.042
*wR*(*F* ^2^) = 0.107
*S* = 1.06
4314 reflections
250 parameters
H-atom parameters constrained
Δρ_max_ = 0.34 e Å^−3^
Δρ_min_ = −0.35 e Å^−3^

### 

Data collection: *CrysAlis CCD* (Oxford Diffraction, 2012 ▶); cell refinement: *CrysAlis CCD*; data reduction: *CrysAlis RED* (Oxford Diffraction, 2012 ▶); program(s) used to solve structure: *SHELXS97* (Sheldrick, 2008 ▶); program(s) used to refine structure: *SHELXL2013* (Sheldrick, 2008 ▶); molecular graphics: *SHELXTL* (Sheldrick, 2008 ▶); software used to prepare material for publication: *publCIF* (Westrip, 2010 ▶).

## Supplementary Material

Crystal structure: contains datablock(s) I. DOI: 10.1107/S1600536814000257/fk2077sup1.cif

Structure factors: contains datablock(s) I. DOI: 10.1107/S1600536814000257/fk2077Isup2.hkl

Supporting information file. DOI: 10.1107/S1600536814000257/fk2077Isup3.mol

Supporting information file. DOI: 10.1107/S1600536814000257/fk2077Isup4.cml

CCDC reference:

Additional supporting information:  crystallographic information; 3D view; checkCIF report

## Figures and Tables

**Fig. 1.**
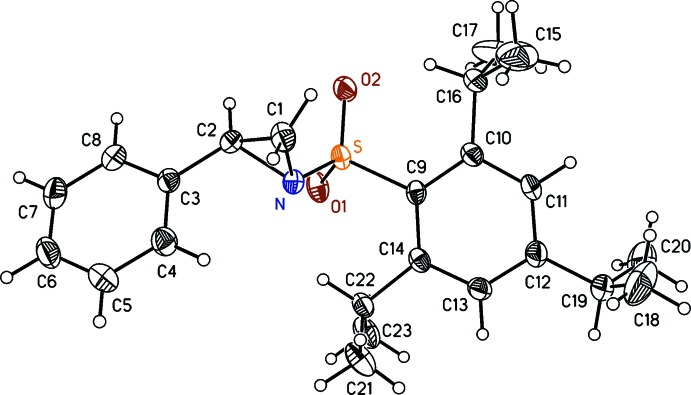
Molecular structure of the title compound with displacement ellipsoids drawn at 50% probability level.

**Fig. 2.**
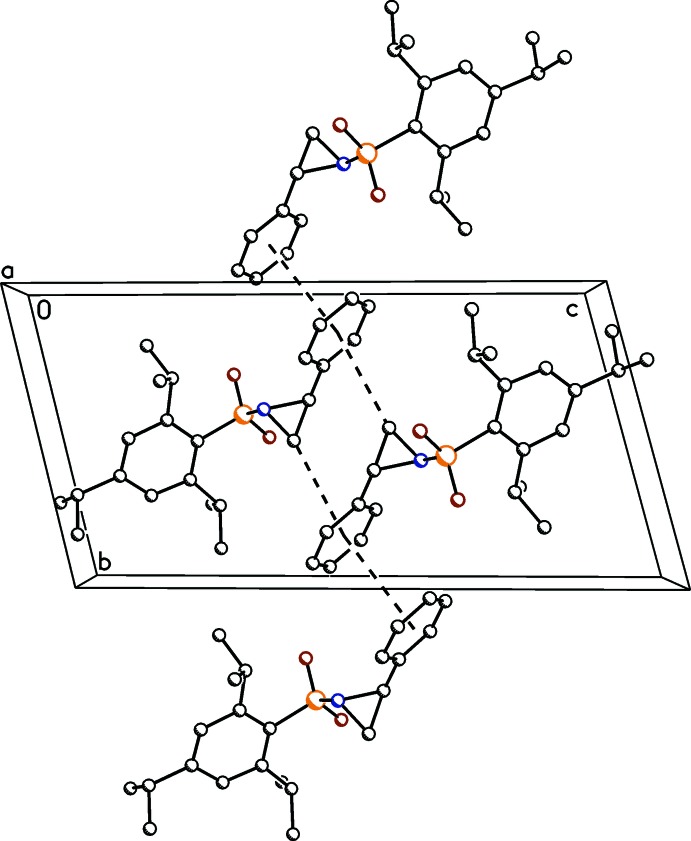
Molecular packing viewed along the a axis with H atoms omitted. Dashed lines indicate π-π-interactions.

## supplementary crystallographic information

### 1. Comment

In the past 10 years, interest in aziridinyl anions generated by direct deprotonation grew steadily (Florio & Luisi, 2010). In our group, deprotonation reactions in *α*- or *β*-position to amines are a frequent research topic (Gessner & Strohmann, 2007; Gessner & Strohmann, 2008*a*,*b*; Unkelbach *et al.*, 2012). We synthesized the compound with the intention to study the deprotonation of the aziridine moiety, which features both the α- and β-position on the same carbon, as well as ringstrain specific effects. The knowledge of the exact structure of the deprotonation substrate is usefull for further discussions of metalleted or substituted derivatives. The succesful deprotonation of such aziridines similar to the title compound were already reported by Huang *et al.*, 2009. The synthesis of the title compound via sulfonium ylide transfer was most recently reportet by Kavanagh *et al.*, 2013.

π—π-Interactions between parallel phenyl groups of adjacent molecules are indicated by a plane-to-plane distance of 3.76 (1) Å, whereas the distance between the isopropyl substituted benzene rings is far longer with 6.30 (1) Å (distance of the centroids). Additionally, interactions between the phenyl ring and the perpendicular aziridine group of an adjacent molecule are possible, with a distance between the *ipso*-carbon (C9) and the aziridine carbon C1 of 3.43 (1) Å. Thus, a T-shaped π-interaction between the aromat and the aziridin moiety are supposable. The C—C-bonds in the triisopropyl substituted benzene ring are not equidistant, but show slight deviations, similar to those found in other structures containing that group (Sandrock *et al.*, 2004, Laba *et al.*, 2009). Longer C—C-bonds were observed at the *ipso*-carbon [C9—C10 1.416 (2) Å and C9—C14 1.415 (2) Å] than for the other [C10—C11 1.396 (2) Å, C11—C12 1.385 (2) Å and C12—C13 1.384 (2) Å]. In addition, C—O-distances [C16—O2 2.83 (1) Å and C22—O1 2.98 (1) Å] shorter than the sum of the corresponding van-der-waals radii suggest strong steric repulsion between the isopropyl groups and the sulfonyl oxygen atoms, causing the sulfur atom to bend out of the aromatic plane (defined by C9-C10-C11-C12-C13-C14) of which it deviates by 0.228 (2) Å.

### 2. Refinement

All H atoms were placed in calculated positions (aromatic C-H = 0.95 Å, primary C-H = 0.98 Å, secondary C-H = 0.99 Å, tertiary C-H = 1.00 Å) and allowed to ride in the refinement with *U_iso_*(H) = 1.2 *U_eq_*(C) and *U_iso_*(H) = 1.5 *U_eq_*(C) for terminal groups.

### Crystal data

**Table:**

C_23_H_31_NO_2_S	*Z* = 2
*M**_r_* = 385.55	*F*(000) = 416
Triclinic, *P*1	*D*_x_ = 1.166 Mg m^−^^3^
*a* = 6.3037 (3) Å	Mo *K*α radiation, λ = 0.71073 Å
*b* = 9.6995 (5) Å	Cell parameters from 5958 reflections
*c* = 18.6675 (9) Å	θ = 2.7–28.8°
α = 75.280 (4)°	µ = 0.16 mm^−^^1^
β = 86.842 (4)°	*T* = 173 K
γ = 84.404 (4)°	Needle, colourless
*V* = 1098.11 (10) Å^3^	0.31 × 0.05 × 0.04 mm

### Data collection

**Table:**

Agilent Xcalibur Sapphire3 diffractometer	4314 independent reflections
Radiation source: Enhance (Mo) X-ray Source	3633 reflections with *I* > 2σ(*I*)
Graphite monochromator	*R*_int_ = 0.045
Detector resolution: 16.0560 pixels mm^-1^	θ_max_ = 26.0°, θ_min_ = 2.3°
φ and ω scans	*h* = −7→7
Absorption correction: multi-scan (*CrysAlis PRO*; Oxford Diffraction, 2012)	*k* = −11→11
*T*_min_ = 0.952, *T*_max_ = 1.000	*l* = −22→22
17550 measured reflections	

### Refinement

**Table:**

Refinement on *F*^2^	Primary atom site location: structure-invariant direct methods
Least-squares matrix: full	Secondary atom site location: difference Fourier map
*R*[*F*^2^ > 2σ(*F*^2^)] = 0.042	Hydrogen site location: inferred from neighbouring sites
*wR*(*F*^2^) = 0.107	H-atom parameters constrained
*S* = 1.06	*w* = 1/[σ^2^(*F*_o_^2^) + (0.0409*P*)^2^ + 0.5494*P*] where *P* = (*F*_o_^2^ + 2*F*_c_^2^)/3
4314 reflections	(Δ/σ)_max_ = 0.001
250 parameters	Δρ_max_ = 0.34 e Å^−^^3^
0 restraints	Δρ_min_ = −0.35 e Å^−^^3^
0 constraints	

### Special details

**Table:**

Experimental. Absorption correction: CrysAlisPro, Agilent Technologies, Version 1.171.36.24 (release 03-12-2012 CrysAlis171 .NET) (compiled Dec 3 2012,18:21:49) Empirical absorption correction using spherical harmonics, implemented in SCALE3 ABSPACK scaling algorithm.
Geometry. All e.s.d.'s (except the e.s.d. in the dihedral angle between two l.s. planes) are estimated using the full covariance matrix. The cell e.s.d.'s are taken into account individually in the estimation of e.s.d.'s in distances, angles and torsion angles; correlations between e.s.d.'s in cell parameters are only used when they are defined by crystal symmetry. An approximate (isotropic) treatment of cell e.s.d.'s is used for estimating e.s.d.'s involving l.s. planes.

### Fractional atomic coordinates and isotropic or equivalent isotropic displacement parameters (Å^2^)

**Table:**

	*x*	*y*	*z*	*U*_iso_*/*U*_eq_	
S	0.05721 (6)	0.42978 (4)	0.83601 (2)	0.02251 (13)	
N	0.2890 (2)	0.41306 (14)	0.87676 (7)	0.0216 (3)	
O1	0.01403 (19)	0.28743 (12)	0.83621 (7)	0.0304 (3)	
O2	−0.10310 (19)	0.51063 (13)	0.87007 (7)	0.0319 (3)	
C1	0.3327 (3)	0.52186 (18)	0.91561 (10)	0.0292 (4)	
H1A	0.2270	0.6057	0.9118	0.035*	
H1B	0.4829	0.5424	0.9172	0.035*	
C2	0.2698 (3)	0.38042 (18)	0.95994 (9)	0.0247 (4)	
H2	0.1206	0.3805	0.9811	0.030*	
C3	0.4221 (3)	0.26355 (17)	1.00030 (9)	0.0228 (4)	
C4	0.6286 (3)	0.24228 (19)	0.97333 (10)	0.0298 (4)	
H4	0.6741	0.3024	0.9277	0.036*	
C5	0.7692 (3)	0.1342 (2)	1.01235 (11)	0.0349 (4)	
H5	0.9114	0.1220	0.9939	0.042*	
C6	0.7035 (3)	0.0440 (2)	1.07802 (11)	0.0382 (5)	
H6	0.7998	−0.0303	1.1047	0.046*	
C7	0.4971 (4)	0.0628 (2)	1.10443 (11)	0.0419 (5)	
H7	0.4508	0.0002	1.1492	0.050*	
C8	0.3560 (3)	0.1726 (2)	1.06619 (10)	0.0322 (4)	
H8	0.2144	0.1853	1.0851	0.039*	
C9	0.1211 (3)	0.52378 (17)	0.74302 (9)	0.0205 (3)	
C10	0.0207 (3)	0.66079 (17)	0.71038 (9)	0.0238 (4)	
C11	0.0609 (3)	0.71662 (18)	0.63479 (9)	0.0282 (4)	
H11	−0.0067	0.8077	0.6116	0.034*	
C12	0.1947 (3)	0.64572 (19)	0.59189 (9)	0.0286 (4)	
C13	0.2961 (3)	0.51463 (18)	0.62672 (9)	0.0272 (4)	
H13	0.3914	0.4663	0.5981	0.033*	
C14	0.2649 (3)	0.45043 (17)	0.70157 (9)	0.0222 (4)	
C15	−0.0578 (5)	0.9059 (2)	0.73340 (15)	0.0609 (7)	
H15A	−0.0836	0.9553	0.6815	0.091*	
H15B	0.0946	0.9011	0.7426	0.091*	
H15C	−0.1392	0.9585	0.7658	0.091*	
C16	−0.1279 (3)	0.75541 (18)	0.74931 (10)	0.0309 (4)	
H16	−0.1170	0.7129	0.8038	0.037*	
C17	−0.3564 (4)	0.7547 (3)	0.73040 (19)	0.0728 (9)	
H17A	−0.3732	0.7958	0.6771	0.109*	
H17B	−0.4484	0.8118	0.7581	0.109*	
H17C	−0.3968	0.6562	0.7436	0.109*	
C18	0.3517 (5)	0.8457 (3)	0.49803 (14)	0.0692 (8)	
H18A	0.2633	0.9177	0.5174	0.104*	
H18B	0.3815	0.8837	0.4450	0.104*	
H18C	0.4862	0.8219	0.5241	0.104*	
C19	0.2340 (3)	0.7118 (2)	0.50981 (10)	0.0397 (5)	
H19	0.3278	0.6407	0.4894	0.048*	
C20	0.0285 (4)	0.7429 (3)	0.46754 (13)	0.0653 (7)	
H20A	−0.0443	0.6551	0.4762	0.098*	
H20B	0.0615	0.7777	0.4144	0.098*	
H20C	−0.0642	0.8160	0.4847	0.098*	
C21	0.6302 (3)	0.3192 (2)	0.71540 (13)	0.0451 (5)	
H21A	0.6587	0.3397	0.6618	0.068*	
H21B	0.7101	0.2292	0.7397	0.068*	
H21C	0.6746	0.3971	0.7342	0.068*	
C22	0.3917 (3)	0.30635 (18)	0.73179 (10)	0.0258 (4)	
H22	0.3678	0.2782	0.7868	0.031*	
C23	0.3133 (4)	0.1912 (2)	0.69989 (12)	0.0403 (5)	
H23A	0.1611	0.1835	0.7123	0.060*	
H23B	0.3933	0.0992	0.7209	0.060*	
H23C	0.3352	0.2168	0.6459	0.060*	

### Atomic displacement parameters (Å^2^)

**Table:**

	*U*^11^	*U*^22^	*U*^33^	*U*^12^	*U*^13^	*U*^23^
S	0.0209 (2)	0.0214 (2)	0.0226 (2)	−0.00145 (16)	−0.00392 (16)	−0.00021 (16)
N	0.0227 (7)	0.0221 (7)	0.0195 (7)	−0.0023 (6)	−0.0030 (6)	−0.0038 (5)
O1	0.0315 (7)	0.0229 (6)	0.0347 (7)	−0.0073 (5)	−0.0106 (5)	0.0006 (5)
O2	0.0262 (7)	0.0321 (7)	0.0301 (7)	0.0046 (5)	0.0039 (5)	0.0017 (5)
C1	0.0376 (10)	0.0233 (9)	0.0285 (9)	−0.0014 (8)	−0.0108 (8)	−0.0080 (7)
C2	0.0295 (9)	0.0259 (9)	0.0188 (8)	0.0015 (7)	−0.0014 (7)	−0.0071 (7)
C3	0.0278 (9)	0.0220 (8)	0.0205 (8)	−0.0016 (7)	−0.0050 (7)	−0.0080 (7)
C4	0.0303 (10)	0.0259 (9)	0.0311 (10)	−0.0029 (7)	−0.0014 (8)	−0.0027 (7)
C5	0.0295 (10)	0.0320 (10)	0.0425 (11)	0.0035 (8)	−0.0042 (8)	−0.0094 (8)
C6	0.0482 (13)	0.0290 (10)	0.0352 (11)	0.0106 (9)	−0.0145 (9)	−0.0066 (8)
C7	0.0572 (14)	0.0366 (11)	0.0245 (10)	0.0046 (10)	−0.0016 (9)	0.0027 (8)
C8	0.0372 (11)	0.0354 (10)	0.0223 (9)	0.0011 (8)	0.0012 (8)	−0.0059 (8)
C9	0.0227 (8)	0.0200 (8)	0.0189 (8)	−0.0029 (6)	−0.0058 (6)	−0.0038 (6)
C10	0.0259 (9)	0.0193 (8)	0.0260 (9)	0.0001 (7)	−0.0035 (7)	−0.0056 (7)
C11	0.0333 (10)	0.0205 (8)	0.0265 (9)	0.0056 (7)	−0.0045 (7)	−0.0003 (7)
C12	0.0339 (10)	0.0282 (9)	0.0219 (9)	0.0017 (8)	−0.0053 (7)	−0.0038 (7)
C13	0.0293 (9)	0.0276 (9)	0.0253 (9)	0.0053 (7)	−0.0040 (7)	−0.0097 (7)
C14	0.0230 (9)	0.0201 (8)	0.0247 (8)	−0.0001 (7)	−0.0083 (7)	−0.0065 (7)
C15	0.0812 (19)	0.0319 (12)	0.0736 (17)	−0.0089 (12)	0.0258 (14)	−0.0251 (12)
C16	0.0393 (11)	0.0205 (9)	0.0295 (9)	0.0048 (8)	0.0035 (8)	−0.0037 (7)
C17	0.0355 (13)	0.084 (2)	0.117 (3)	0.0111 (13)	−0.0003 (14)	−0.0659 (19)
C18	0.087 (2)	0.0712 (18)	0.0413 (14)	−0.0245 (15)	0.0178 (13)	0.0033 (12)
C19	0.0517 (13)	0.0381 (11)	0.0227 (9)	0.0129 (9)	0.0010 (9)	−0.0022 (8)
C20	0.0744 (18)	0.0837 (19)	0.0292 (12)	0.0079 (15)	−0.0155 (12)	−0.0012 (12)
C21	0.0329 (11)	0.0404 (12)	0.0580 (14)	0.0097 (9)	−0.0171 (10)	−0.0066 (10)
C22	0.0306 (9)	0.0219 (9)	0.0253 (9)	0.0054 (7)	−0.0095 (7)	−0.0073 (7)
C23	0.0517 (13)	0.0244 (10)	0.0470 (12)	0.0056 (9)	−0.0197 (10)	−0.0126 (9)

### Geometric parameters (Å, º)

**Table:**

S—O1	1.4322 (12)	C13—C14	1.389 (2)
S—O2	1.4382 (13)	C13—H13	0.9500
S—N	1.6597 (14)	C14—C22	1.530 (2)
S—C9	1.7880 (16)	C15—C16	1.518 (3)
N—C1	1.478 (2)	C15—H15A	0.9800
N—C2	1.504 (2)	C15—H15B	0.9800
C1—C2	1.489 (2)	C15—H15C	0.9800
C1—H1A	0.9900	C16—C17	1.503 (3)
C1—H1B	0.9900	C16—H16	1.0000
C2—C3	1.486 (2)	C17—H17A	0.9800
C2—H2	1.0000	C17—H17B	0.9800
C3—C4	1.385 (2)	C17—H17C	0.9800
C3—C8	1.389 (2)	C18—C19	1.519 (3)
C4—C5	1.384 (3)	C18—H18A	0.9800
C4—H4	0.9500	C18—H18B	0.9800
C5—C6	1.382 (3)	C18—H18C	0.9800
C5—H5	0.9500	C19—C20	1.519 (3)
C6—C7	1.378 (3)	C19—H19	1.0000
C6—H6	0.9500	C20—H20A	0.9800
C7—C8	1.390 (3)	C20—H20B	0.9800
C7—H7	0.9500	C20—H20C	0.9800
C8—H8	0.9500	C21—C22	1.528 (3)
C9—C14	1.415 (2)	C21—H21A	0.9800
C9—C10	1.416 (2)	C21—H21B	0.9800
C10—C11	1.396 (2)	C21—H21C	0.9800
C10—C16	1.531 (2)	C22—C23	1.525 (2)
C11—C12	1.385 (2)	C22—H22	1.0000
C11—H11	0.9500	C23—H23A	0.9800
C12—C13	1.384 (2)	C23—H23B	0.9800
C12—C19	1.520 (2)	C23—H23C	0.9800
			
O1—S—O2	116.59 (8)	C9—C14—C22	125.91 (15)
O1—S—N	105.66 (7)	C16—C15—H15A	109.5
O2—S—N	111.07 (7)	C16—C15—H15B	109.5
O1—S—C9	108.62 (7)	H15A—C15—H15B	109.5
O2—S—C9	111.50 (7)	C16—C15—H15C	109.5
N—S—C9	102.29 (7)	H15A—C15—H15C	109.5
C1—N—C2	59.91 (11)	H15B—C15—H15C	109.5
C1—N—S	118.58 (11)	C17—C16—C15	112.2 (2)
C2—N—S	113.79 (11)	C17—C16—C10	111.28 (16)
N—C1—C2	60.94 (10)	C15—C16—C10	111.45 (16)
N—C1—H1A	117.7	C17—C16—H16	107.2
C2—C1—H1A	117.7	C15—C16—H16	107.2
N—C1—H1B	117.7	C10—C16—H16	107.2
C2—C1—H1B	117.7	C16—C17—H17A	109.5
H1A—C1—H1B	114.8	C16—C17—H17B	109.5
C3—C2—C1	124.07 (16)	H17A—C17—H17B	109.5
C3—C2—N	115.58 (13)	C16—C17—H17C	109.5
C1—C2—N	59.15 (10)	H17A—C17—H17C	109.5
C3—C2—H2	115.3	H17B—C17—H17C	109.5
C1—C2—H2	115.3	C19—C18—H18A	109.5
N—C2—H2	115.3	C19—C18—H18B	109.5
C4—C3—C8	119.08 (16)	H18A—C18—H18B	109.5
C4—C3—C2	121.46 (15)	C19—C18—H18C	109.5
C8—C3—C2	119.45 (16)	H18A—C18—H18C	109.5
C5—C4—C3	120.59 (17)	H18B—C18—H18C	109.5
C5—C4—H4	119.7	C20—C19—C18	111.1 (2)
C3—C4—H4	119.7	C20—C19—C12	111.87 (18)
C6—C5—C4	120.28 (18)	C18—C19—C12	110.91 (17)
C6—C5—H5	119.9	C20—C19—H19	107.6
C4—C5—H5	119.9	C18—C19—H19	107.6
C7—C6—C5	119.43 (18)	C12—C19—H19	107.6
C7—C6—H6	120.3	C19—C20—H20A	109.5
C5—C6—H6	120.3	C19—C20—H20B	109.5
C6—C7—C8	120.63 (18)	H20A—C20—H20B	109.5
C6—C7—H7	119.7	C19—C20—H20C	109.5
C8—C7—H7	119.7	H20A—C20—H20C	109.5
C3—C8—C7	119.96 (18)	H20B—C20—H20C	109.5
C3—C8—H8	120.0	C22—C21—H21A	109.5
C7—C8—H8	120.0	C22—C21—H21B	109.5
C14—C9—C10	121.29 (15)	H21A—C21—H21B	109.5
C14—C9—S	116.84 (12)	C22—C21—H21C	109.5
C10—C9—S	121.69 (12)	H21A—C21—H21C	109.5
C11—C10—C9	117.04 (15)	H21B—C21—H21C	109.5
C11—C10—C16	116.07 (14)	C23—C22—C21	111.22 (16)
C9—C10—C16	126.88 (15)	C23—C22—C14	110.57 (14)
C12—C11—C10	123.22 (15)	C21—C22—C14	110.51 (15)
C12—C11—H11	118.4	C23—C22—H22	108.1
C10—C11—H11	118.4	C21—C22—H22	108.1
C13—C12—C11	117.74 (16)	C14—C22—H22	108.1
C13—C12—C19	121.16 (16)	C22—C23—H23A	109.5
C11—C12—C19	121.07 (16)	C22—C23—H23B	109.5
C12—C13—C14	123.04 (16)	H23A—C23—H23B	109.5
C12—C13—H13	118.5	C22—C23—H23C	109.5
C14—C13—H13	118.5	H23A—C23—H23C	109.5
C13—C14—C9	117.56 (15)	H23B—C23—H23C	109.5
C13—C14—C22	116.53 (15)		

### Deviation of atoms from the benzene ring least-squares plane (Å).

**Table:**

Atom	Deviation
C9	0.020 (1)
C10	-0.013 (1)
C11	-0.005 (1)
C12	0.015 (1)
C13	-0.009 (1)
C14	-0.009 (1)
S1*	0.228 (2)

Note: (*) not used in the least-squares-plane calculation.
